# Structural Analysis of a Heteropolysaccharide from *Saccharina japonica* by Electrospray Mass Spectrometry in Tandem with Collision-Induced Dissociation Tandem Mass Spectrometry (ESI-CID-MS/MS)

**DOI:** 10.3390/md10102138

**Published:** 2012-09-25

**Authors:** Weihua Jin, Jing Wang, Sumei Ren, Ni Song, Quanbin Zhang

**Affiliations:** 1 Institute of Oceanology, Chinese Academy of Science, Qingdao 266071, China; Email: jwh.054130305@yahoo.com.cn (W.J.); jingwang@qdio.ac.cn (J.W.); 2 University of Chinese Academy of Sciences, Beijing 100049, China; 3 Nantong Marine Science and Technology R & D Center, IOCAS, Jiangsu 226006, China; 4 College of Medicine and Pharmaceutics, Ocean University of China, Qingdao 266003, China; Email: rensumei@ouc.edu.cn (S.R.); nisong1975@ouc.edu.cn (N.S.)

**Keywords:** ESI-CID-MS/MS, fucoidan, oligosaccharide, *Saccharina japonica*

## Abstract

A fucoidan extracted from *Saccharina japonica* was fractionated by anion exchange chromatography. The most complex fraction F0.5 was degraded by dilute sulphuric acid and then separated by use of an activated carbon column. Fraction Y1 was fractionated by anion exchange and gel filtration chromatography while Fraction Y2 was fractionated by gel filtration chromatography. The fractions were determined by ESI-MS and analyzed by ESI-CID-MS/MS. It was concluded that F0.5 had a backbone of alternating 4-linked GlcA and 2-linked Man with the first Man residue from the nonreducing end accidentally sulfated at C6. In addition, F0.5 had a 3-linked glucuronan, in accordance with a previous report by NMR. Some other structural characteristics included GlcA 1→3 Man 1→4 GlcA, Man 1→3 GlcA 1→4 GlcA, Fuc 1→4 GlcA and Fuc 1→3 Fuc. Finally, it was shown that fucose was sulfated at C2 or C4 while galactose was sulfated at C2, C4 or C6.

## 1. Introduction

Fucoidan, which is a family of sulfated heteropolysaccharides extracted from brown algae and invertebrates, has been increasingly studied because of its biological activity, including antitumor activity [1,2], protective effects against γ-radiation-induced blood cell damage [3], antiangiogenic activity [4], immunodulating activity [5], inhibition of colony formation in human melanoma and colon cancer cells [6] and the most studied anticoagulant activity [7,8,9,10,11]. However, the relationships between fucoidan structure and biological activity are poorly understood due to the complexity of fucoidan structure. Research on structural features has traditionally been conducted using chemical methods, such as methylation analysis and NMR [7,9,12]; however, structural characteristics are still largely unknown. It has been reported that fucoidan contains two different main backbones, one made up of (1→3)-linked fucopyranose residues with fucose branches [12] and another with repeating (1→3) and (1→4) glycosidic bonds [13]. It was also demonstrated that the positions of the sulfate groups and of the branching units, including fucose residues, sulfated fucose residues, galactose residues and glucuronic acid residues, were different. These results are similar to previous studies [14,15].

Sulfated and fucosylated glucuronomannan was also found in *Kjellmaniella crassifolia* and investigated by Sakai *et al.* [16]. Later, it was reported [17] that fucoidan extracted from *Hizikia fusiforme* was composed of →2) α-D-Man (1→ and →4) β-D-GlcA (1→ alternatively while slight →4) β-D-Gal (1→ was mixed in the main chain. The branched points were at C-3 of →2) α-D-Man (1→, C-2 of →4) β-D-Gal (1→ and C-2 of →6) β-D-Gal (1→. About 2/3 of fucose were at the nonreducing ends, and left of them were 1→4, 1→3 and 1→2 glycosidic linkages. About 2/3 of xylose were at the nonreducing ends, and left of them were 1→4 glycosidic linkage. It was sulfated at C-6 of →2,3) α-D-Man (1→, C-4 and C-6 of →2) α-D-Man (1→, C-3 of →6) β-D-Gal (1→, C-2, C-3 or C-4 of fucose, while some fucose had two sulfate groups. In addition, Bilan *et al.* [12] also found the existence of glucuronomannan in *Saccharina latissima*. 

However, mass spectrometry (MS) applied to the structural analysis of saccharides has allowed the determination of minor structural components. Along with NMR spectroscopy, electrospray mass spectrometry (ESI) and Matrix-Assisted Laser Desorption/Ionization Time of Flight mass spectrometry (MALDI-TOF) have become primary analytical tools, providing high sensitivity and selectivity. In addition, electrospray mass spectrometry in tandem with collision-induced dissociation tandem mass spectrometry (ESI-CID-MS/MS) has yielded information on the type of linkages, sulfation, and the backbone sequences of saccharides. Through electrospray ion trap mass spectrometry and capillary electrophoresis, it has been possible to differentiate the three isomers 2-O-, 3-O- and 4-O-sulfated fucose [18]. Finally, oligosaccharide mixtures derived from sulfated carrageenan-derived oligosaccharides [19] and fucoidan of *Ascophyllum nodosum* [20], *Fucus evanescens* [13] and *Laminaria cichorioides* [21] have been analyzed by ESI-CID-MS/MS to elucidate the structural features of oligosaccharides. Recently, it was reported [22] that a fucoidan isolated from Hizikia fusiforme was analyzed by partial acid hydrolysis followed by characterization of the oligosaccharide fragments using ESI-CID-MS/MS.

This study is also dedicated to structural characteristics of fucoidan extracted from *Saccharina japonica *using NMR, ESI-MS and ESI-MS/MS.

## 2. Results and Discussion

### 2.1. Preparation of Fucoidan and Oligosaccharides

**Figure 1:**
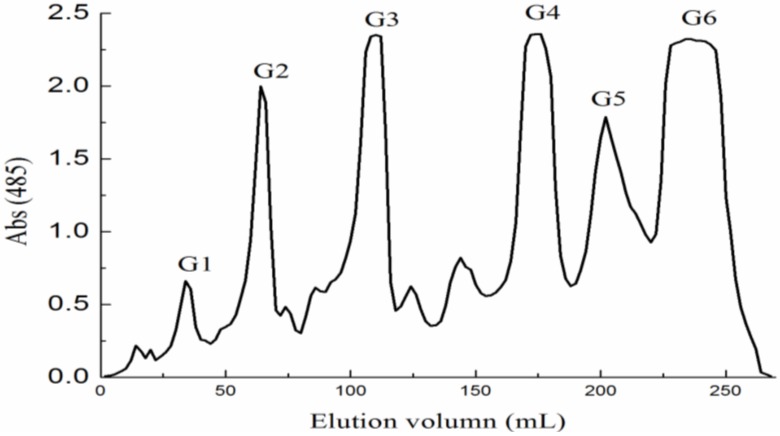
Gel filtration chromatography of Y2 oligosaccharide on a Bio-Gel P-4 Gel column.

In this paper, we separate three fractions. With respect to monosaccharide and sulfate content (Table 1), the first fraction (F0.5) contained a large amount of uronic acid and a small amount of sulfate while the subsequent two fractions (F1 and F2) contained trace amounts of uronic acid and large amounts of sulfate. In other words, F0.5 consisted mainly of sulfated fucomannoglucuronan whereas F1 and F2 consisted mainly of sulfated galactofucan and sulfated fucan. To further study its structure, F0.5 was degraded with 4% sulphuric acid, and the resulting mixture separated by 95% ethanol precipitation and ran through the activated carbon column to yield two main fractions, Y1 and Y2. Y1 was fractioned by anion exchange chromatography to produce three fractions: YF (water), YD (0.05 M NaCl) and YT (0.1 M NaCl). Fraction YD and YT were purified by gel filtration chromatography with an elution of water (Supplementary data). YD had two peaks named YD-1 and YD-2 while YT showed a symmetric peak. In addition, Y2 was presumed to be a mixture of neutral oligosaccharides, it was immediately fractionated by gel filtration chromatography to yield six fractions: G1–G6 (Figure 1). The molecular weight and degree of polymerization (DP) of all fractions were determined by negative-ion ESI-MS. The apparent structural composition of each fraction is summarized in Table 2.

**Table 1:** Chemical composition (%, dry weight) of the degraded polysaccharides.

Sample	Fuc (%)	U A (%)	SO4 (%)	Monosaccharides (molar ratio)							Mw
				Fuc	Gal	Man	Glc A	Rha	Xyl	Glc	
F0.5	13.77	20.34	29.07	1	0.98	0.80	0.95	0.12	0.30	0.40	5954
F1	54.84	7.3	32.26	1	0.36	0.13	0.10	0.02	0.04	0.10	8436
F2	35.04	0.71	53.40	1	0.07	0.03	0.01	0	0	0.02	12586
YF	-	-	-	1	0.43	0.32	0.28	0.41	0	0.26	-
YD-1	-	-	-	0	1.10	11.14	12.59	0.17	0	1.08	-
YD-2	-	-	-	1	0.28	3.53	1.96	0	0	0.29	-
YT	-	-	-	0	0	8.21	9.30	0	0	1.48	-
G5	-	-	-	1	0.11	0.02	0	0	0	0.07	-
G6	-	-	-	1	0.04	0	0	0	0	0.05	-

**Table 2:** Structural compositions of all fractions separated from degraded F0.5.

Samples		DP	Ions (charges)	*m/z*	Predicted structural compositions
YF		1	193.035(−1)	193.035	GlcA
		2	355.086(−1)	355.086	GlcAMan
		2	369.065(−1)	369.065	GlcA_2_
		2	339.091(−1)	339.091	GlcAFuc
		3	531.117(−1)	531.117	GlcA_2_Man
		4	346.081(−2)	693.170	GlcA_2_Man_2_
			693.170(−1)		
		6	515.123(−2)	1031.249	GlcA_3_Man_3_
YD	YD-1	2	184.032(−2)	369.065	GlcA_2_
			369.065(−1)		
		2	217.018(−2)	435.030	GlcAManSO_3_H
		3	265.055(−2)	531.117	GlcA_2_Man
			531.117(−1)		
		4	346.081(−2)	693.170	GlcA_2_Man_2_
			693.170(−1)		
		4	386.059(−2)	773.117	GlcA_2_Man_2_SO_3_H
		6	515.123(−2)	1031.249	GlcA_3_Man_3_
	YD-2	1	193.035(−1)	193.035	GlcA
		1	243.017(−1)	243.017	FucSO_3_H
		1	259.012(−1)	259.012	GalSO_3_H
		2	339.231(−1)	339.231	GlcAFuc
		2	355.086(−1)	355.086	GlcAMan
		2	217.072(−2)	435.033	GlcAManSO_3_H
YT		2	217.072(−2)	435.033	GlcAManSO_3_H
		3	257.037(−3)	545.096	GlcA_3_
		3	305.033(−2)	611.068	GlcA_2_ManSO_3_H
		4	386.059(−2)	773.118	GlcA_2_Man_2_SO_3_H
		5	474.073(−2)	949.147	GlcA_3_Man_2_SO_3_H
		6	369.731(−3)	1111.201	GlcA_3_Man_3_SO_3_H
			555.101(−2)		
G1		8	1369.333(−1)	1369.333	GlcA_4_Man_4_
G2		6	343.081(−3)	1031.249	GlcA_3_Man_3_
			515.125(−2)		
			1031.249(−1)		
G3		4	346.082(−2)	693.173	GlcA_2_Man_2_
			693.173(−1)		
G4		2	355.086(−1)	355.086	GlcAMan

### 2.2. Analysis of the Oligosaccharides of All Fractions by ESI-MS

**Figure 2:**
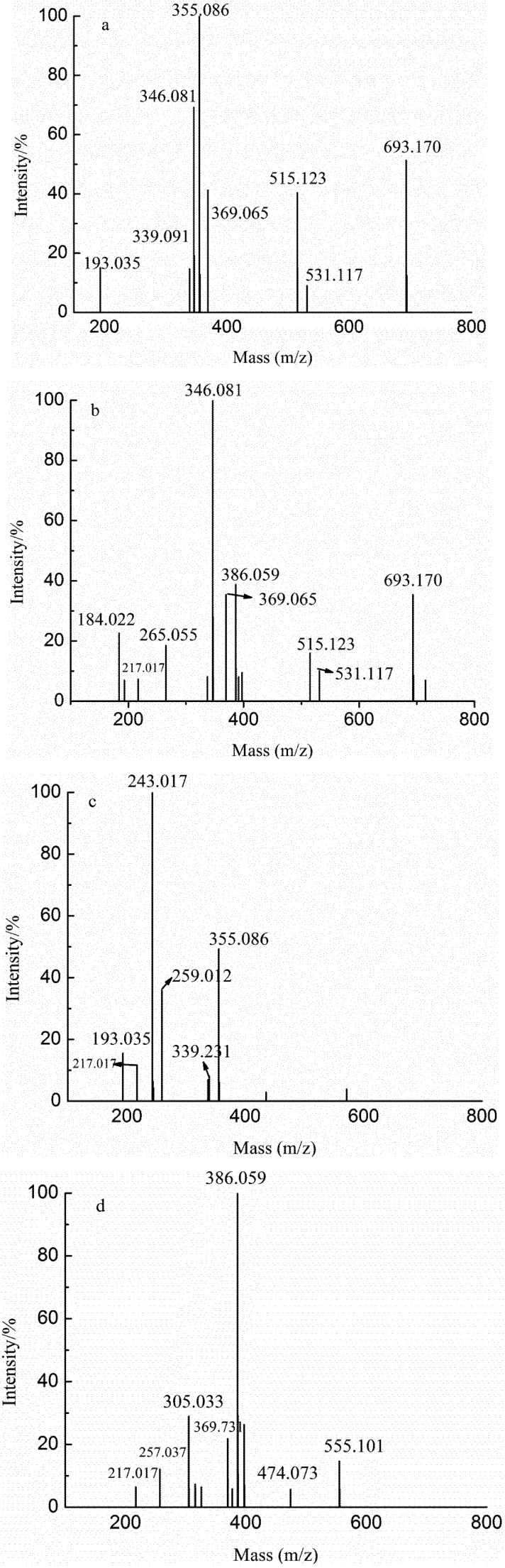
Negative-ion mode electrospray mass spectrometry (ESI-MS) spectra of YF (**a**), YD-1 (**b**), YD-2 (**c**) and YT (**d**).

YF was obtained by anion exchange chromatography on a DEAE-Bio Gel Agarose FF gel with elution by water. Thus YF was a mixture of neutral oligosaccharides, which was approved by the results in Table 2 and Figure 2a.

In the ESI-MS spectrum of YD-1 (Figure 2b), there were six apparent structural compositions (GlcA_2_, GlcAManSO_3_H, GlcA_2_Man, GlcA_2_Man_2_, GlcA_2_Man_2_SO_3_H and GlcA_3_Man_3_) corresponding to the ions at *m/z* 184.032 (−2)/369.065 (−1), 217.017 (−2), 265.055 (−2)/531.117 (−1), 346.081 (−2)/693.170 (−1), 386.059 (−2) and 515.123 (−2), respectively. Six ions at *m/z* 193.035, 243.017, 259.012, 339.231, 355.086 and 435.033 were detected in the ESI-MS spectrum of YD-2 (Figure 2c), suggesting the presence of GlcA, FucSO_3_H, GalSO_3_H, GlcAFuc, GlcAMan and GlcAManSO_3_H, respectively. Inference of structural composition was based on the analysis of the molar ratio of monosaccharide (Table 1). Interestingly, the main fraction of YD-2 was FucSO_3_H while the main fraction of YD-1 was GlcA_2_Man_2_; these data might suggest that the eluting power of 0.05 M NaCl (Fraction YD) was equivalent to the substances containing one sulfate group (such as FucSO_3_H) or two uronic acid residues (such as GlcA_2_Man_2_).

Our data further suggested that the eluting power of 0.1 M NaCl (Fraction YT) was equivalent to the substances consisting of two sulfate groups (such as Fuc(SO_3_H)_2_), one sulfate group and two uronic acid residues (GlcA_2_ManSO_3_H) or four uronic acid residues (GlcA_4_). In the ESI-MS spectrum of fraction YT, the main ion at *m/z* 386.059 (−2) (Figure 2d) was identified as GlcA_2_Man_2_SO_3_H while the less intensive ions detected at *m/z* 217.017 (−2), 257.037 (−3), 305.033 (−2), 386.059 (−2), 474.073 (−2), 369.731 (−3)/555.101 (−2) corresponded to GlcAManSO_3_H, GlcA_3_, GlcA_2_ManSO_3_H, GlcA_2_Man_2_SO_3_H, GlcA_3_Man_2_SO_3_H, and GlcA_3_Man_3_SO_3_H, respectively. These results helped us to confirm the above speculation. The absorptive capacity of the DEAE-Bio Gel Agarose FF gel permitted detection of ions at *m/z* 386.059 (−2) (Figure 2b,d).

**Figure 3:**
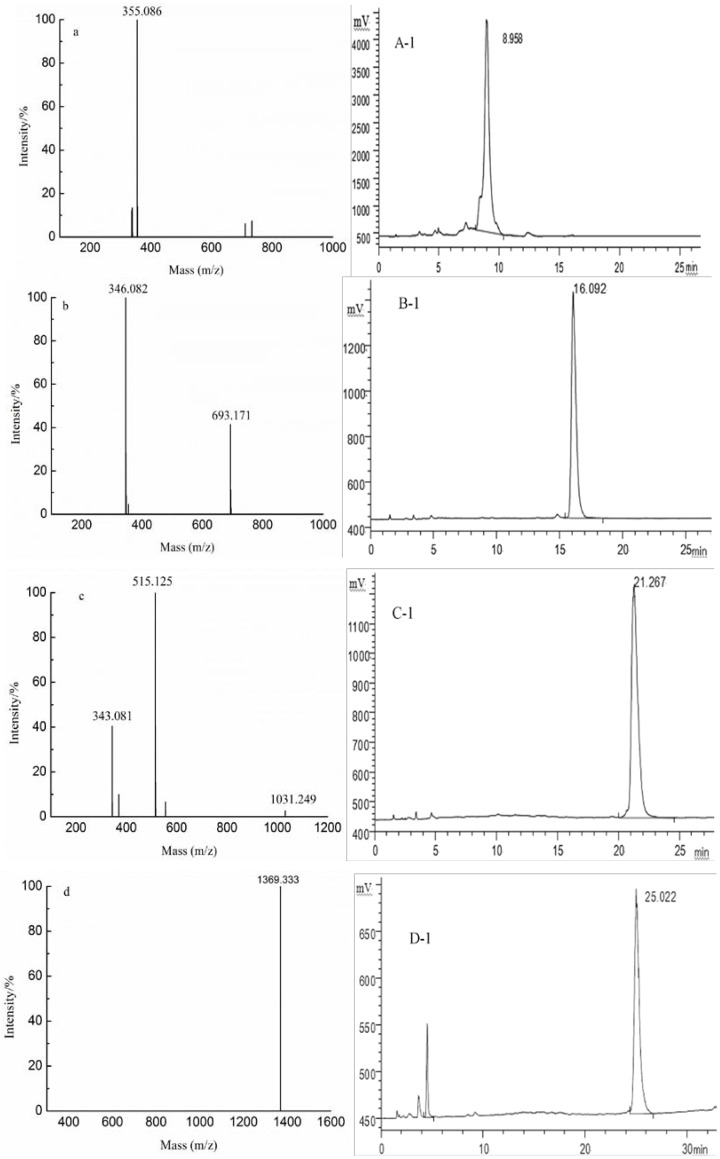
Negative-ion mode ESI-MS spectra and HPLC spectra of G1 (**d**,**D-1**), G2 (**c**,**C-1**), G3 (**b**,**B-1**) and G4 (**a**,**A-1**).

Y2 was fractionated by gel filtration chromatography to obtain six fractions (G1–G6). The results (Figure 3) of HPLC analysis confirmed that G1, G2, G3 and G4 were relatively pure. Fractions G1, G2, G3 and G4 were identified by NMR (supplementary data) as a series of oligosaccharides consisted of a repeating disaccharide unit of GlcA and Man. The degrees of polymerization (DP) of each fraction were 8, 6, 4 and 2, respectively (Figure 3). The results suggest that the bonds between mannose and glucuronic acid were stable against the acid condition and that F0.5 had a backbone of –[Man-GlcA]*_n_*–. Owing to the separation of gel filtration chromatography, it was indicated that Fraction G5 consisted of disaccharides and monosaccharides while G6 consisted mainly of fucose and a smaller quantity of galactose and glucose (Table 1). To further study the exact structural features of F0.5, some samples were subjected to ESI-CID-MS/MS.

### 2.3. Analysis of the Structural Features of the Fractions by ESI-CID-MS/MS

The ^0,2^X (*m/z* 138.970) and ^0,2^A (*m/z* 182.996) ions indicated sulfation of Fuc at C2 or C4 in the ESI-CID-MS/MS spectrum of the ion at *m/z* 243.017 (not shown), whereas the ^0,2^A (*m/z* 198.991), ^2,5^A (*m/z* 180.980) and ^0,3^A (*m/z* 168.981) ions suggested sulfation of Gal at C4 or at C6 in the ESI-CID-MS/MS spectrum of the ion at *m/z* 259.011 (not shown). 

**Figure 4:**
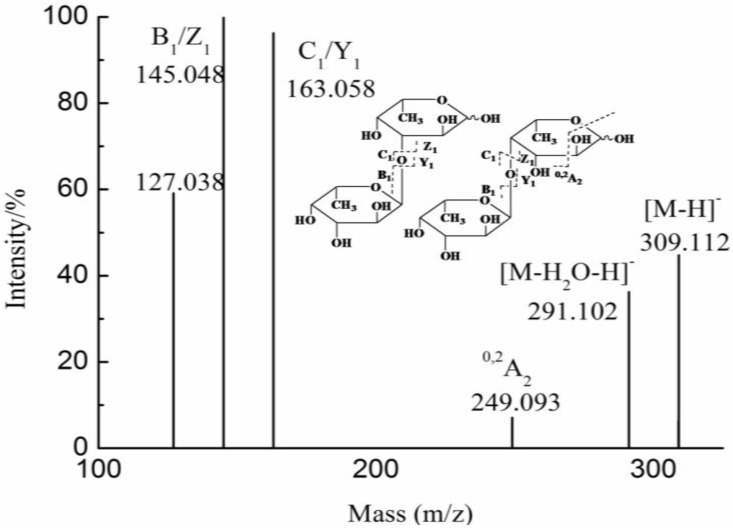
Negative-ion mode electrospray mass spectrometry in tandem with collision-induced dissociation tandem mass spectrometry (ESI-CID-MS/MS) spectrum of the ion at *m/z* 309.112.

In fraction G5, the ion at *m/z* 309.112 (Figure 4), corresponding to [Fuc_2_ − H]^−^ (supplementary data), was detected. The ions at *m/z* 145.048 and 163.058 were assigned as B_1_/Z_1 _and C_1_/Y_1_, respectively. The low intensity fragmentation ion at *m/z* 249.093 was ^0,2^A_2_, suggesting the prevalence of 1→3 linkage between the two Fuc residues and minor presence of 1→4 linkage. This has been confirmed by previous study [15]. Therefore, the ion (*m/z* 309.112), corresponded to disaccharide, was major Fuc 1→3 Fuc and minor Fuc 1→4 Fuc.

**Figure 5:**
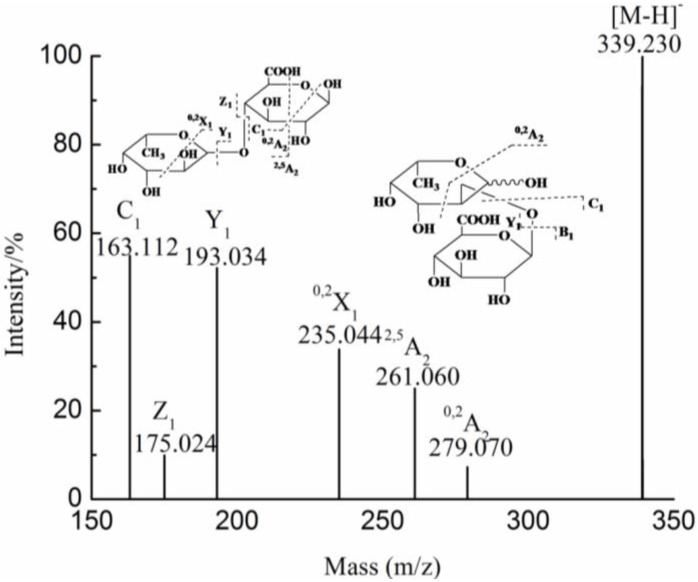
Negative-ion mode ESI-CID-MS/MS spectrum of the ion at *m/z* 339.230.

The ion detected at *m/z* 339.230 (Figure 5) confirmed that the oligosaccharides with the GlcA residue were more stable in the acid condition. The C_1_ (*m/z* 163.112) and Y_1_ (193.034) ions corresponded to Fuc and GlcA residues, respectively. The characteristic ion at *m/z* 235.044, assigned to the ^0,2^X_1_ ion, suggested that the reducing terminal was a GlcA residue. Therefore, the ion at *m/z* 339.230 was Fuc-GlcA. In addition, the ^2,5^A_2_ (*m/z* 261.060) and ^0,2^A_2_ (*m/z* 279.070) ions indicated that the linkage between the Fuc and GlcA residues was a 1→4 linkage. Moreover, we could also speculate that the ions at *m/z* 163.112 (Y_1_), 175.024 (B_1_), 193.034 (C_1_) and 235.044 (^0,2^A_2_) suggested that the structural feature of the ion at *m/z* 339.230 was GlcA 1→2 Fuc.

**Figure 6:**
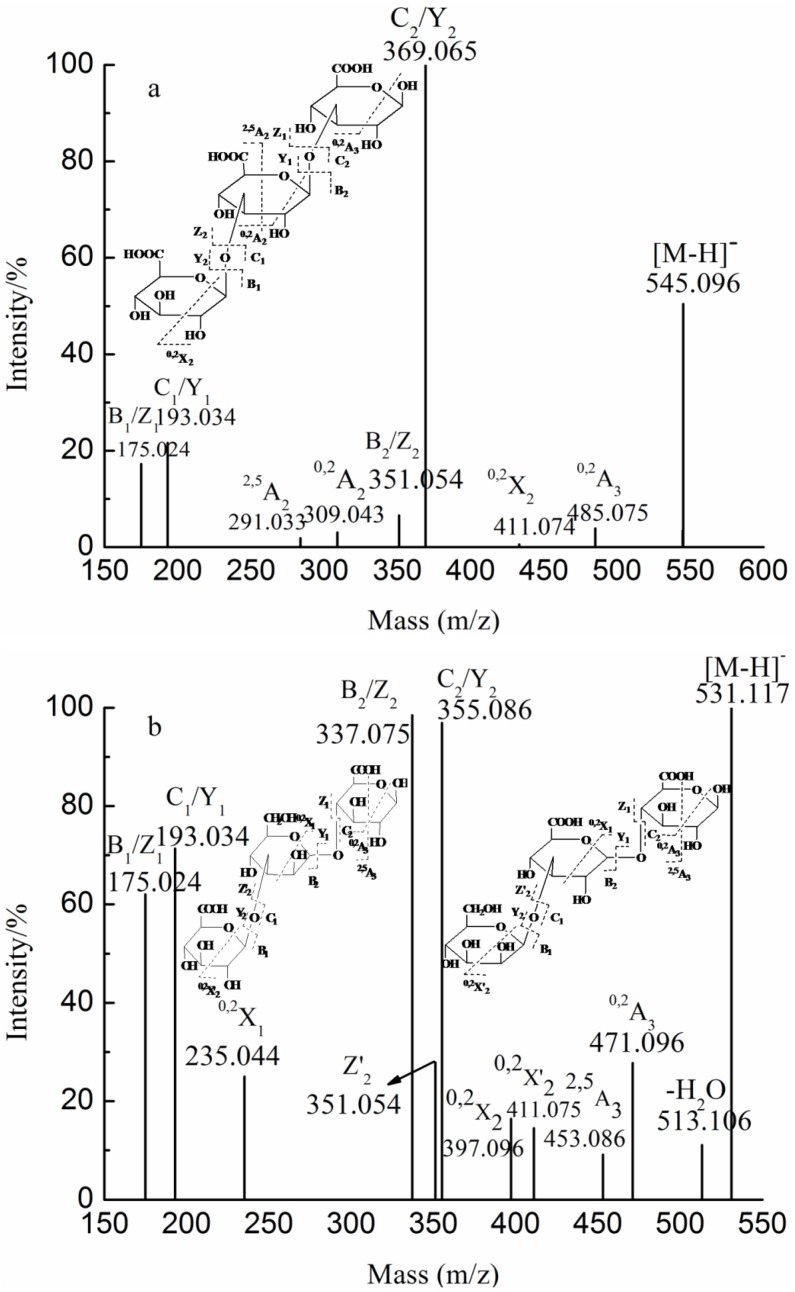
Negative-ion mode ESI-CID-MS/MS spectra of the ions at *m/z* 545.096 (**a**) and 531.117 (**b**).

The ions at *m/z* 193.035, 184.032 (−2)/369.065 (−1) and 257.037 (−3) corresponded to GlcA, GlcA_2_ and GlcA_3_, suggesting the presence of glucuronan. To elucidate the fine structure of glucuronan, the ion at *m/z* 545.096 was analyzed by ESI-CID-MS/MS (Figure 6a). Four series of B/C-type and Y/Z-type ions suggested that glucuronan was a trisaccharide with GlcA-GlcA-GlcA. The low intensity characteristic ions at *m/z* 309.043 and 485.075, corresponding to ^0,2^A_2_ and ^0,2^A_3_ ions, respectively, indicated the prevalence of a 3-linked GlcA. These data suggest a backbone of 1→3 linked glucuronan.

The analyses of glucuronomannan and sulfated glucuronomannan suggest that the linkage of 1→4 was more sensitive in the acid condition than that of linkage of 1→2. However, we also detected the less intensive ion at *m/z* 531.117 (Figure 6b), assigned as [GlcA_2_Man − H]^−^. The ions at *m/z* 175.024 and 193.034 are indicative of dehydrated GlcA and GlcA residues from the nonreducing or reducing end, corresponding to the B_1_/C_1_-type or Z_1_/Y_1_-type ions, respectively. The ^0,2^X_1_-type ion suggests that the reducing end consisted of a GlcA residue. However, we found two ^0,2^X_2_-type ions at *m/z* 397.096 and 411.075, named ^0,2^X_2_ and ^0,2^X_2_, respectively, which suggests two structural sequences of trisaccharide. As the ^0,2^X_1_-type ion suggests a reducing end of GlcA residue, we conclude that one sequence was Man-GlcA-GlcA and the other was GlcA-Man-GlcA. The identity of the former was confirmed by the presence of the ion at *m/z* 351, corresponding to a Z_2_-type ion. The ions at *m/z* 337.075 and 355.086 were identified as B_2_/Z_2_-type and C_2_/Y_2_-type ions, respectively. The characteristic ions at *m/z* 453.086 and 471.096 corresponded to ^2,5^A_3_-type and ^0,2^A_3_-type ions, respectively, suggesting that the two residues from reducing terminal had a 1→4 glycosidic bond. ^0,2^A_2_-type ions were not found, indicating that the two residues from the nonreducing terminal had a 1→3 glycosidic bond. In summary, the two structural sequences of trisaccharide were Man 1→3 GlcA 1→4 GlcA and GlcA 1→3 Man 1→4 GlcA.

**Figure 7:**
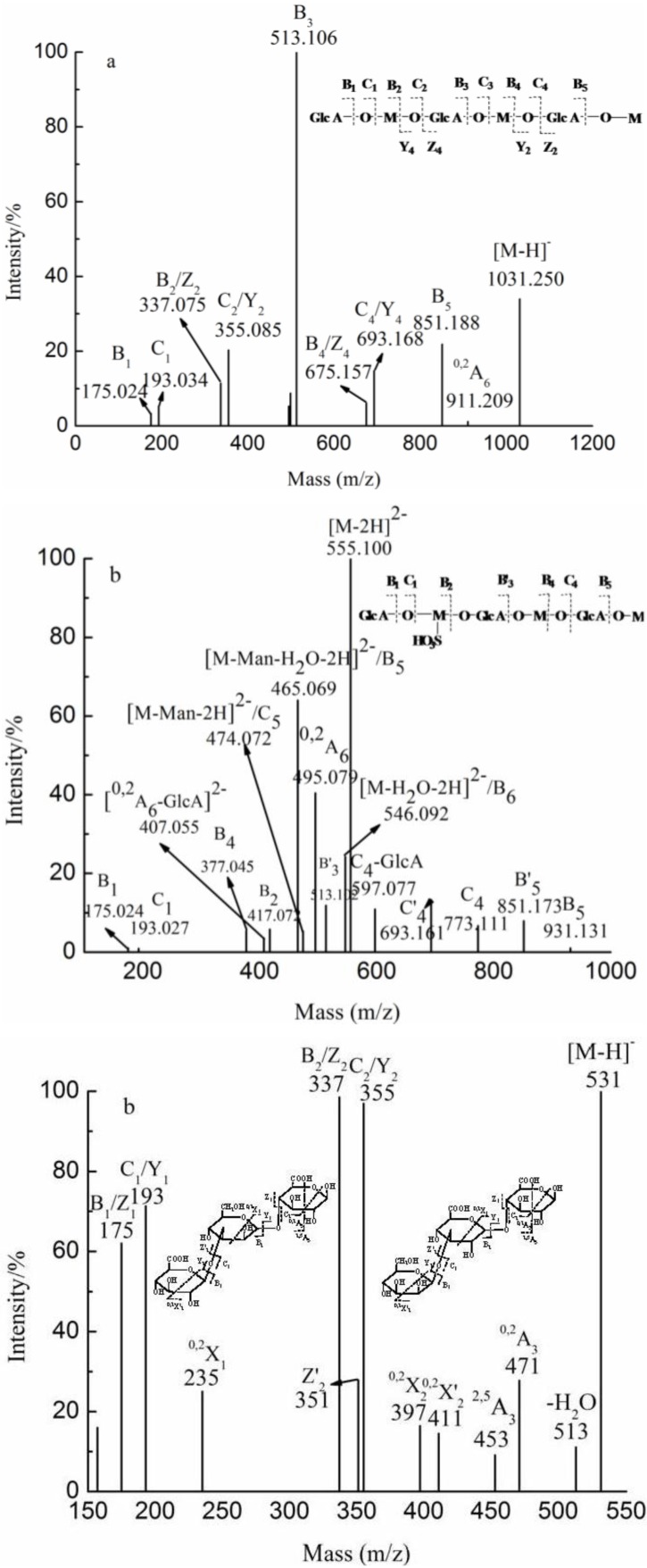
Negative-ion mode ESI-CID-MS/MS spectra of the ions at *m/z* 1031.250 (**a**) and 555.100 (−2) (**b**).

The fragmentation pattern of ions at *m/z* 355.086, 346.082 (−2) /693.173 (−1) and 1369.333 (not shown) were similar to that of the ion at *m/z* 343.081 (−3)/ 515.125 (−2)/ 1031.249 (−1) (Figure 7a). The fragment ion at *m/z* 1031 corresponded to [GlcA_4_Man_4_ − H]^−^. The ions at *m/z* 175.024 and 193.249 were the characteristic fragments, corresponding to fragment B_1_ ion and C_1_ ion, respectively. The results suggest that the nonreducing terminal was a GlcA residue. Two series of B-type and C-type ions from the nonreducing end were found with Y-type and Z-type ions from the reducing end; this pattern confirmed a linear backbone of alternating GlcA and Man. In addition, the characteristic ions of ^0,2^A-type were detected. However, the ions at *m/z* 235 and 573 do not appear in (Figure 7a) because of their lower intensity. The ^0,2^A-type [23] ions were the cross-ring cleavage by releasing the C1-C2 portion of the reducing-ring, leading to the loss of C_4_H_8_O_4 _at *m/z* 120. However, no characteristic ion of ^0,2^A-trpe was detected. And there was no information obtained regarding the linkage of GlcA residue in the spectra of ESI-CID-MS/MS. The linkage of GlcA residue was therefore determined by NMR (supplementary data) and confirmed the results of ESI-CID-MS/MS. Finally, it was concluded that F0.5 had a backbone of repeating 4-linked GlcA and 2-linked Man, in accordance with Bilan *et al.* [12] and Sakai *et al.* [16].

Bilan *et al.* [12] also reported the presence of sulfated glucuronomannan. YT was primarily composed of a sulfated tetrasaccharide. The ion at *m/z* 555.100 (−2) corresponded to [GlcA_3_Man_3_SO_3_H − H]^−^ (Figure 7b). The ions at *m/z* 175.024 and 193.034 were identified as dehydrated GlcA and GlcA residues, suggesting that the nonreducing end was GlcA residue. The reducing terminal was identified as Man based on the presence of ions at *m/z* 931.131/465.069 (−2) and 474.072 (−2), assigned as B_5_ and C_5_, respectively. A series of B-type ions at *m/z* 175.024 (B_1_), 417.022 (B_2_), 377.045 (−2) (B_4_), 931.131 (B_5_) along with C-type ions at *m/z* 193.034 (C_1_), 773.111 (C_4_) indicated that the linear sequence was GlcA-ManSO_3_H-GlcA-Man-GlcA-Man. The ion at *m/z* 259.011 (ManSO_3_H) was also confirmed in the above analysis. The ions at *m/z* 513.102 (B’_3_), 693.161 (C’_4_) and 851.173 (B’_5_) suggested that the sulfate group was lost whereas the ions at *m/z* 407.055 [(^0,2^A_6_-GlcA)^2−^] and 597.077 (C_4_-GlcA) suggested that the GlcA residue from the nonreducing end was sensitive in the negative mode. The characteristic ion at *m/z* 495.079 (−2) was detected, suggesting that the glycosidic bond between the GlcA and Man residues was 1→2 linkage. However, the ion at *m/z* 326 (−2) did not appear due to its low intensity. Although the linkage of the Man residue was identified as a 2-linked Man, the linkage of the GlcA residue was not determined by ESI-CID-MS/MS. With the assistances of NMR (supplementary data) and the above results, the linkage of the GlcA residue was determined to be a 4-linked GlcA and the substitution of sulfation of Man was at C6. 

## 3. Experimental Section

### 3.1. Materials

The brown algae *S. japonica *was obtained from Shazikou, Qingdao, China in May 2011. Seven standard monosaccharides (L-fucose (Fuc), D-galactose (Gal), D-mannose (Man/M), D-glucuronic acid (GlcA), L-rhamnose monohydrate (Rha), D-xylose (Xyl) and D-glucose (Glc)) were purchased from Sigma-Aldrich. 3-Methyl-1-phenyl-2-pyrazolin-5-one (99%) was purchased from Aldrich chemistry. 

### 3.2. Preparation and Purification of Fucoidans

Crude fucoidan was extracted following Wang *et al.* [14] and degraded using hydrogen dioxide and ascorbic acid to obtain a fucoidan of low molecular weight. Briefly speaking, crude fucoidan (1 g) was dissolved in water (100 mL). Then ascorbic acid (0.5 g) and hydrogen dioxide (0.3 mL) were added and the solution was stirred for 1 h at room temperature. Then, it was ultrafiltrated, concentrated and precipitated by 72% ethanol before further precipitation with 95% ethanol. Finally, the degraded polysaccharides were dried by using an infrared lamp. The degraded polysaccharides (60 g) underwent anion exchange chromatography on a DEAE-Bio Gel Agarose FF gel (12 cm × 70 cm) with elution by 0.5 M (35L) (F0.5), 1 M (35L) (F1) and 2 M NaCl (35L) (F2). The polysaccharides were then ultrafiltrated, concentrated and precipitated by 72% ethanol before further precipitation with 95% ethanol. Finally, the F0.5, F1 and F2 factions were dried by using an infrared lamp.

### 3.3. Preparation and Purification of Oligosaccharides from F0.5

Polysaccharide F0.5 was dissolved with reflux in 4% sulphuric acid (60 mg mL^−1^) for 5 h and then neutralized with barium hydroxide after cooling to room temperature. The solution was centrifugated and the supernate was concentrated. The concentrated solution was fractionated using the activated carbon column (2.6 cm × 30 cm) with a gradient elution from 50% ethanol to 95% ethanol. Then, eluent (Y1) was concentrated and precipitated in 95% ethanol. The activated carbon column was washed with a gradient elution from 50% ethanol to 95% ethanol. The elution (Y2) was combined, concentrated and freeze-dried. Y1 (1.0 g) was separated by anion exchange chromatography on a DEAE-Bio Gel Agarose FF gel (2.6 cm × 30 cm) with elution by water (YF), 0.05 M NaCl (YD) and 0.1 M NaCl (YT). The concentrated solutions of fractions YD and YT were desalted and separated on a Sephadex G-10 column (2.6 cm × 100 cm) with an elution of water. Y2 (0.5 g) was separated on a Bio-Gel P-4 Gel (Extra Fine, <45 μM) column (2.6 cm × 100 cm) (Figure 1) eluted with 0.5 M NH_4_HCO_3_ at a flow rate 0.14 mL min^−1^. It was collected every 14 min per pipe after 27 h. Six fractions were collected and lyophilized.

### 3.4. Composition Analysis

The sulfated content was determined by ion chromatography on Shodex IC SI-52 4E column (4.0 × 250 mm) eluted with 3.6 mM Na_2_CO_3 _at a flow rate of 0.8 mL min^−1^ at 45 °C. The molar ratio of monosaccharide composition and the content of fucose were determined following Zhang *et al.* [24]. Briefly speaking, a solution of sample (10 mg mL^−1^) was hydrolyzed in 2 M trifluoroacetic acid in a 10 mL ampoule. The ampoule was sealed in a nitrogen atmosphere and hydrolyzed for 4 h at 110 °C. Then the hydrolyzed mixture was neutralized to pH 7 with sodium hydroxide. Later the mixture was converted into its 1-phenyl-3-methyl-5-pyrazolone derivatives and separated by HPLC chromatography. Uronic acid was analyzed by a modified carbazole method [25]. Molecular weight was determined by GPC-HPLC on TSK gel PWxl 3000 column (7 μm 7.8 × 300 mm) eluted with 0.2 M Na_2_SO_4_ at a flow rate of 0.5 mL min^−1^ at 30 °C.

### 3.5. MS Analysis of Oligosaccharides

ESI-MS was performed on a Micromass Q-Tof Ultima instrument (Waters, Manchester, UK).

Samples were dissolved in CH_3_CN-H_2_O (1:1, v/v). Mass spectra were registered in the negative ion mode at a flow rate of 5 μL min^−^^1^. The capillary voltage was set to −3000 V, and the cone voltage was set at −50 V. The source temperature was 80 °C, and the desolvation temperature was 150 °C. The collision energy was optimized between 10 and 50 eV. All spectra were analyzed by MassLynx software.

### 3.6. Condition of HPLC

The fractions were analyzed by HPLC with an ELSD detector, performed on a “click” maltose column (10 μm, 10 × 150 mm) at 1 mL min^−1^ in a gradient solution. Gradient 1: 0–30 min, water-acetonitrile-ammonium formate buffer (100 mM, pH 3.0): 10:80:10 (v/v/v)→40:50:10(v/v/v). Gradient 2: 30–35 min, water-acetonitrile-ammonium formate buffer (100 mM, pH 3.0): 40:50:10 (v/v/v)→75:15:10(v/v/v).

## 4. Conclusions

To further determine the precise structure of F0.5, it was degraded with partial acid hydrolysis (due to the absence of available enzymes). The depolymerized mixture was then separated by use of an activated carbon column. Y1 was purified using anion exchange and gel filtration chromatography while Y2 was purified using gel filtration chromatography. The results suggest that F0.5 consisted of two types of polysaccharides: (1) a glucuronomannan and a sulfated glucuronomannan with the same backbone of repeating 4-linked GlcA and 2-linked Man, and the latter with the first mannopyranose residue from the nonreducing terminus sulfated at C-6 and (2) a glucuronan with a backbone of 3-linked GlcA. There were also some other structural fragments, including GlcA 1→3 Man 1→4 GlcA, Man 1→3 GlcA 1→4 GlcA, Fuc 1→4 GlcA and Fuc 1→3 Fuc. Combined with the results obtained in this study and a previous study [15], it was concluded that it might provide the whole structural model of fucoidan.

## Supplementary Files

Supplementary File 1: PDF-Document (PDF, 473 KB)
