# Supplementary material for: Structural Analysis of a Heteropolysaccharide from Saccharina japonica by Electrospray Mass Spectrometry in Tandem with Collision-Induced Dissociation Tandem Mass Spectrometry (ESI-CID-MS/MS)
Source: Mar Drugs. 2012 Sep 25;10(10):2138–52. doi: 10.3390/md10102138 (PMC3497013; doi:10.3390/md10102138)

Supporting Information

Table of Contents

Figure S1. Gel filtration chromatography of **YD** on a Sephadex-G10 Gel column. 2
Figure S2. Gel filtration chromatography of **YT** on a Sephadex-G10 Gel column. 3
Figure S3. ^{1}H-NMR of **G2**. 4
Figure S4. DEPTQ (C-NMR) of **G2**. 5
Figure S5. HMBC spectrum of **G2**. 6
Figure S6. 2D HSQC spectrum of **G2**. 7
Figure S7. 2D TOCSY of **G2**. 8
Figure S8. ^{1}H-NMR of **G3**. 9
Figure S9. DEPTQ (C-NMR) of **G3**. 10
Figure S10. ^{1}H-NMR of **G3**. 11
Figure S11. DEPTQ (C-NMR) of **G3**. 12
Figure S12. ^{1}H-NMR of **YT**. 13
Figure S13. DEPTQ (C-NMR) of **YT**. 14

Figure S1. Gel filtration chromatography of **YD** on a Sephadex-G10 Gel column.

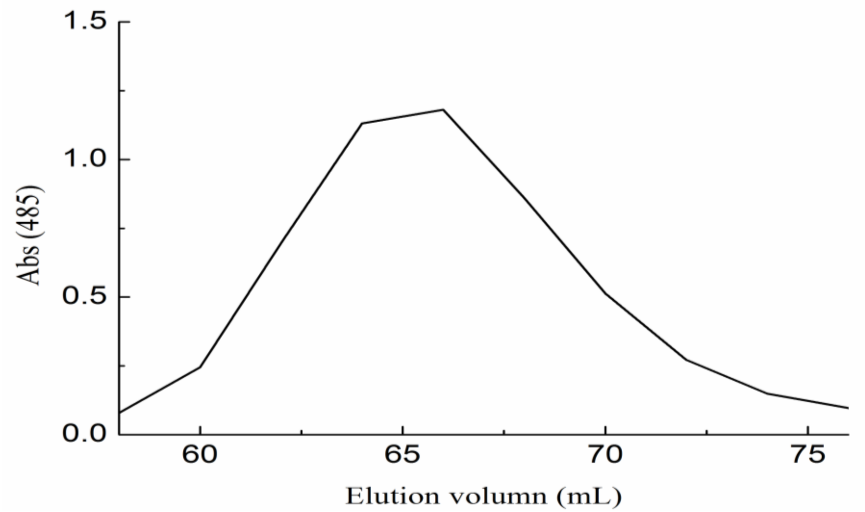

Figure S2. Gel filtration chromatography of **YT** on a Sephadex-G10 Gel column.

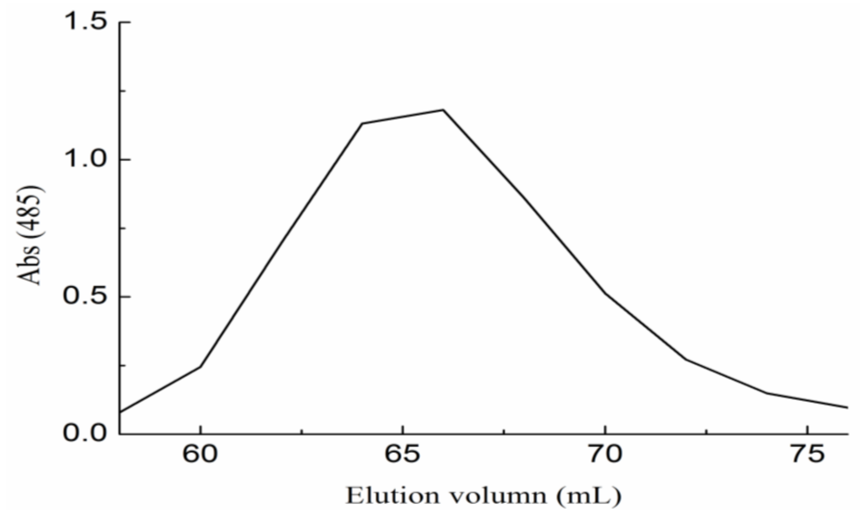

Figure S3. ^{1}H-NMR of **G2**.

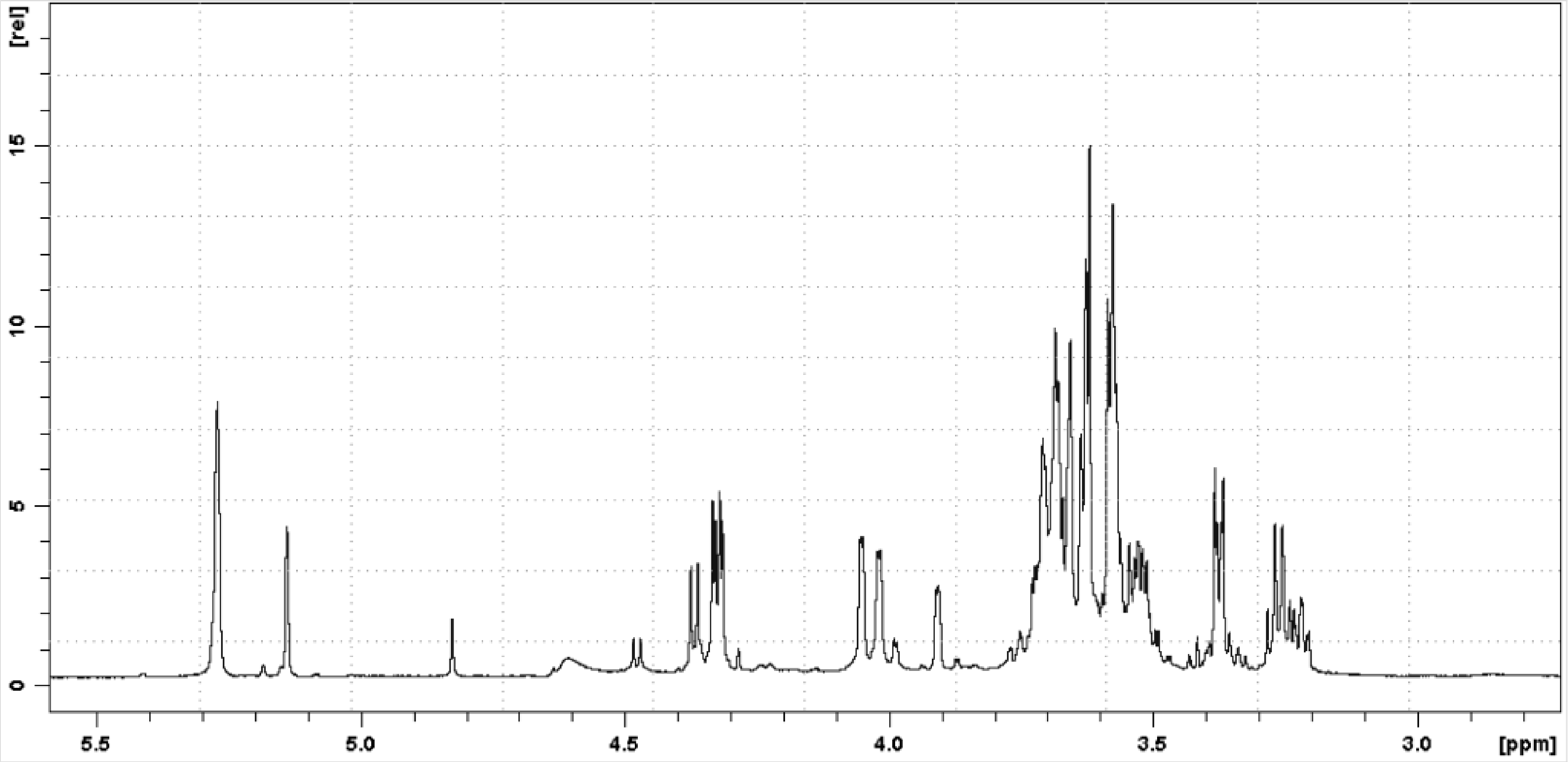

Figure S4. DEPTQ (C-NMR) of **G2**.

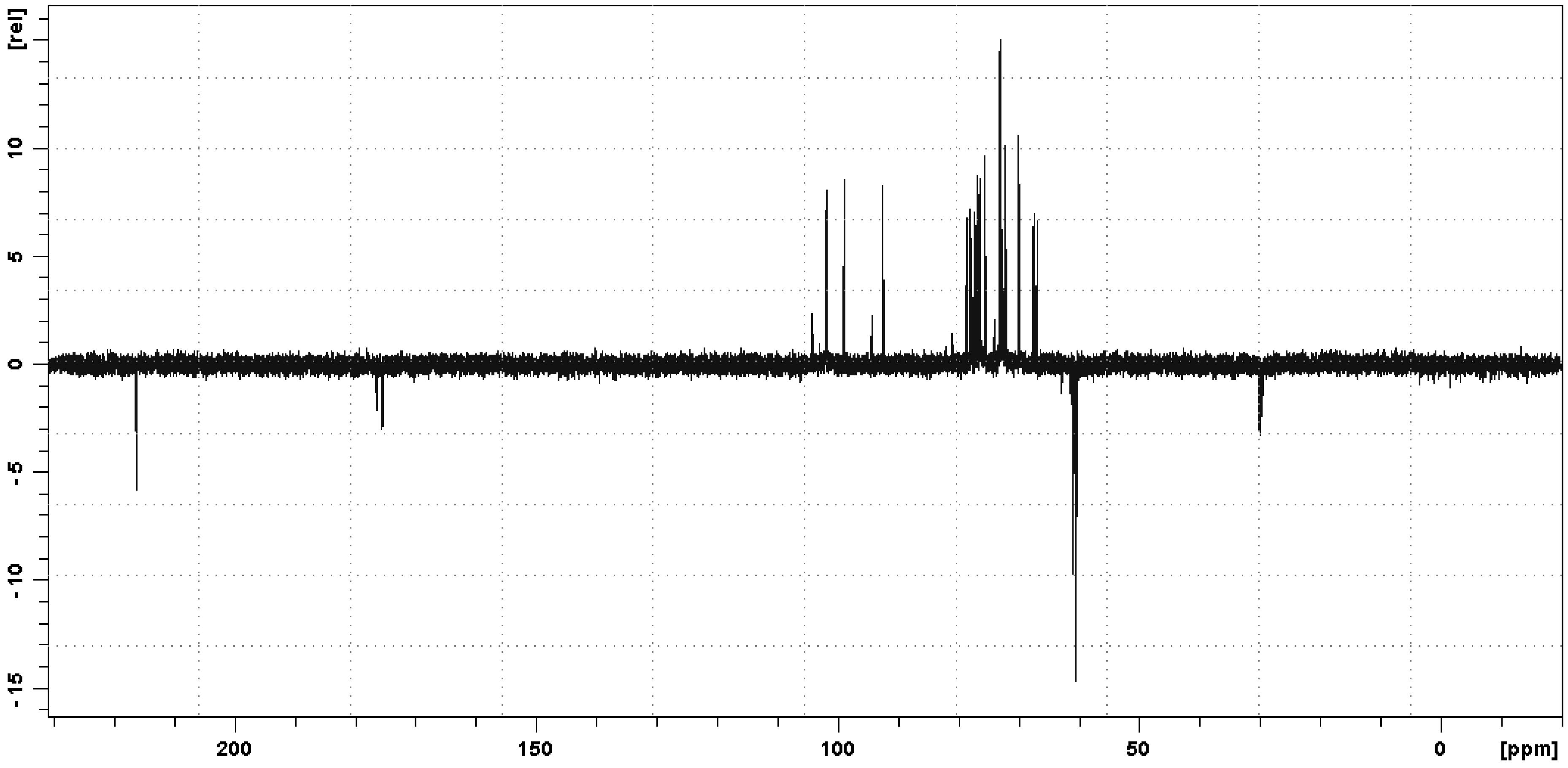

Figure S5. HMBC spectrum of **G2**.

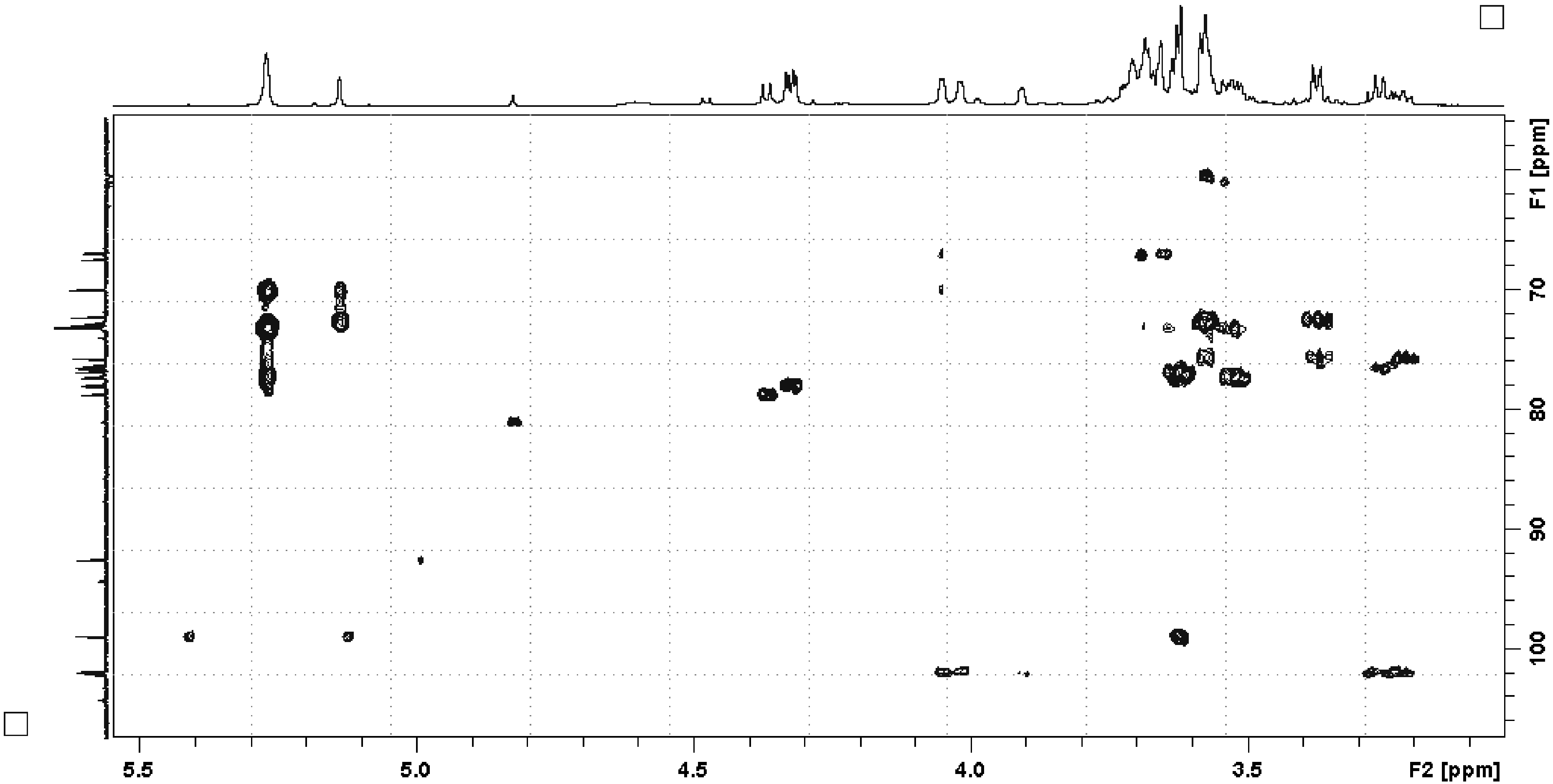

Figure S6. 2D HSQC spectrum of **G2**.

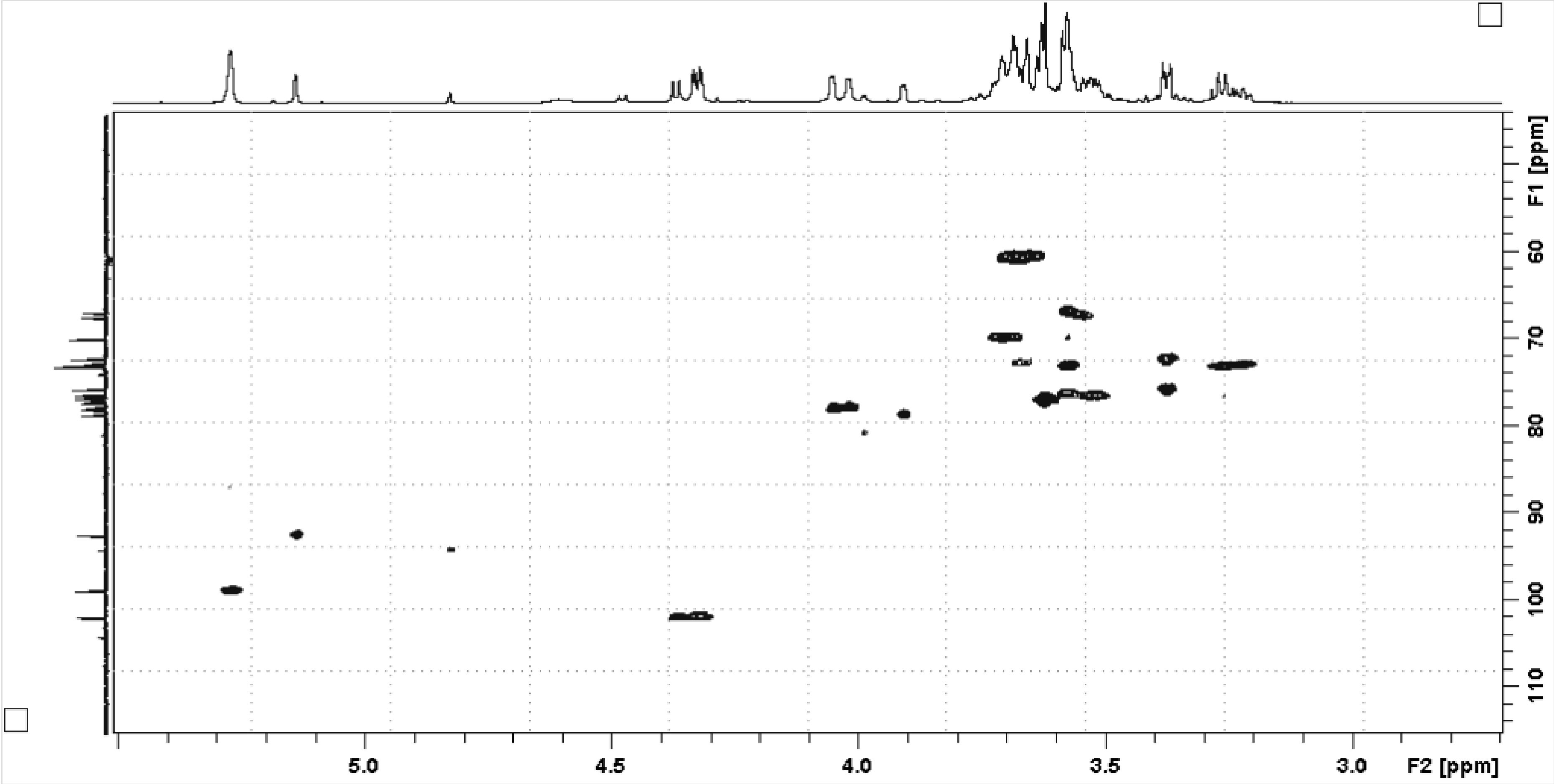

Figure S7. 2D TOCSY of **G2**.

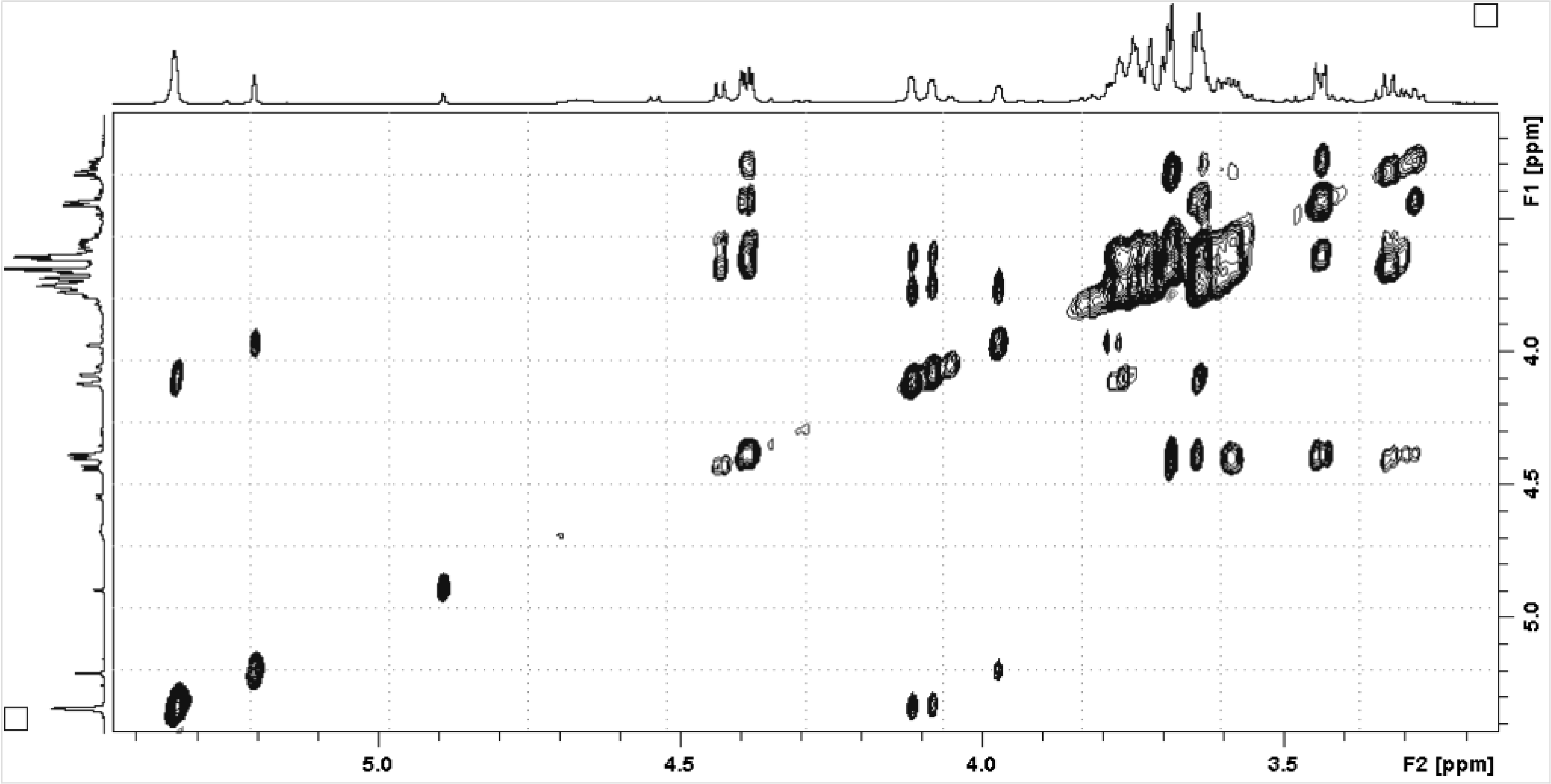

Figure S8. ^{1}H-NMR of **G3**.

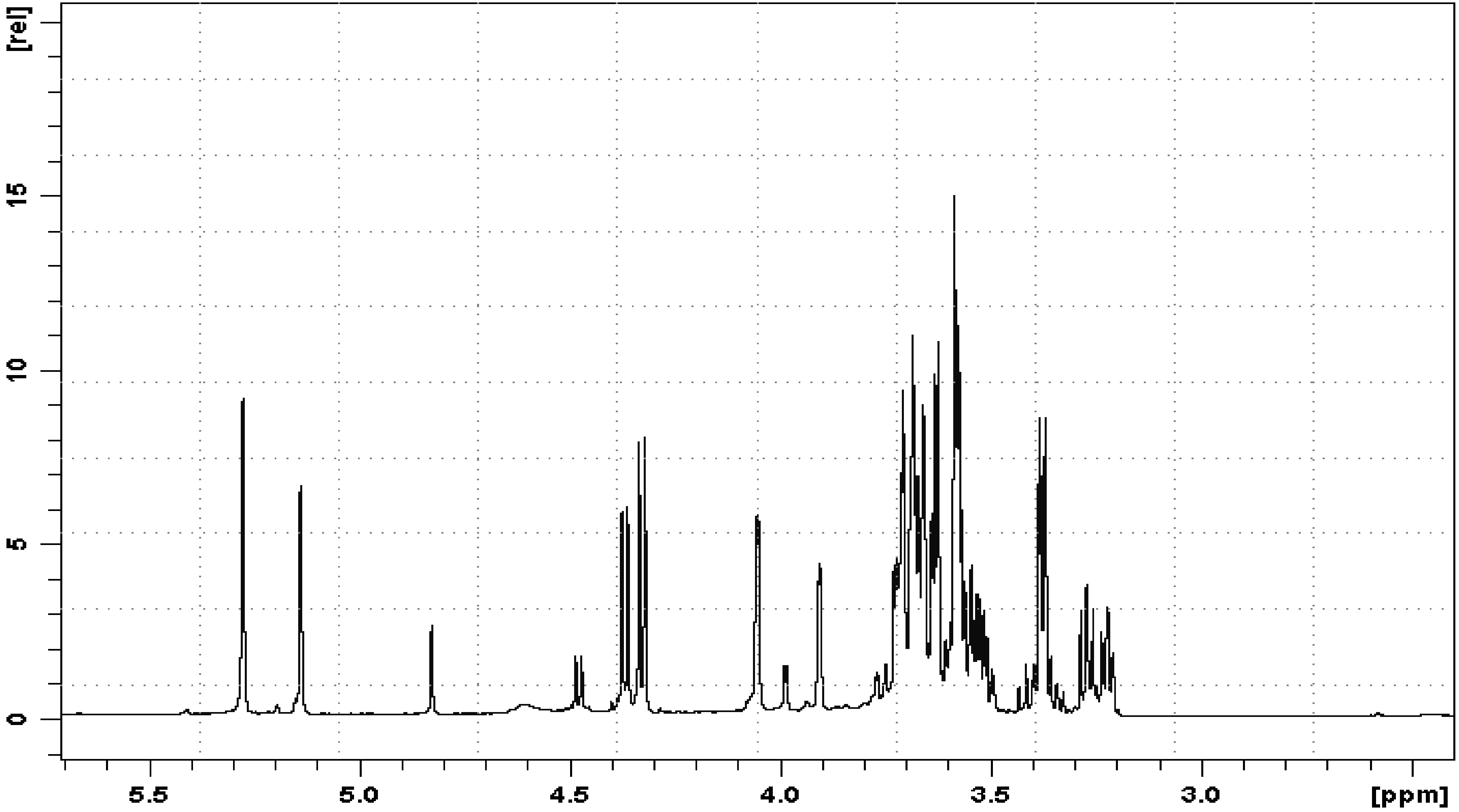

Figure S9. DEPTQ (C-NMR) of **G3**.

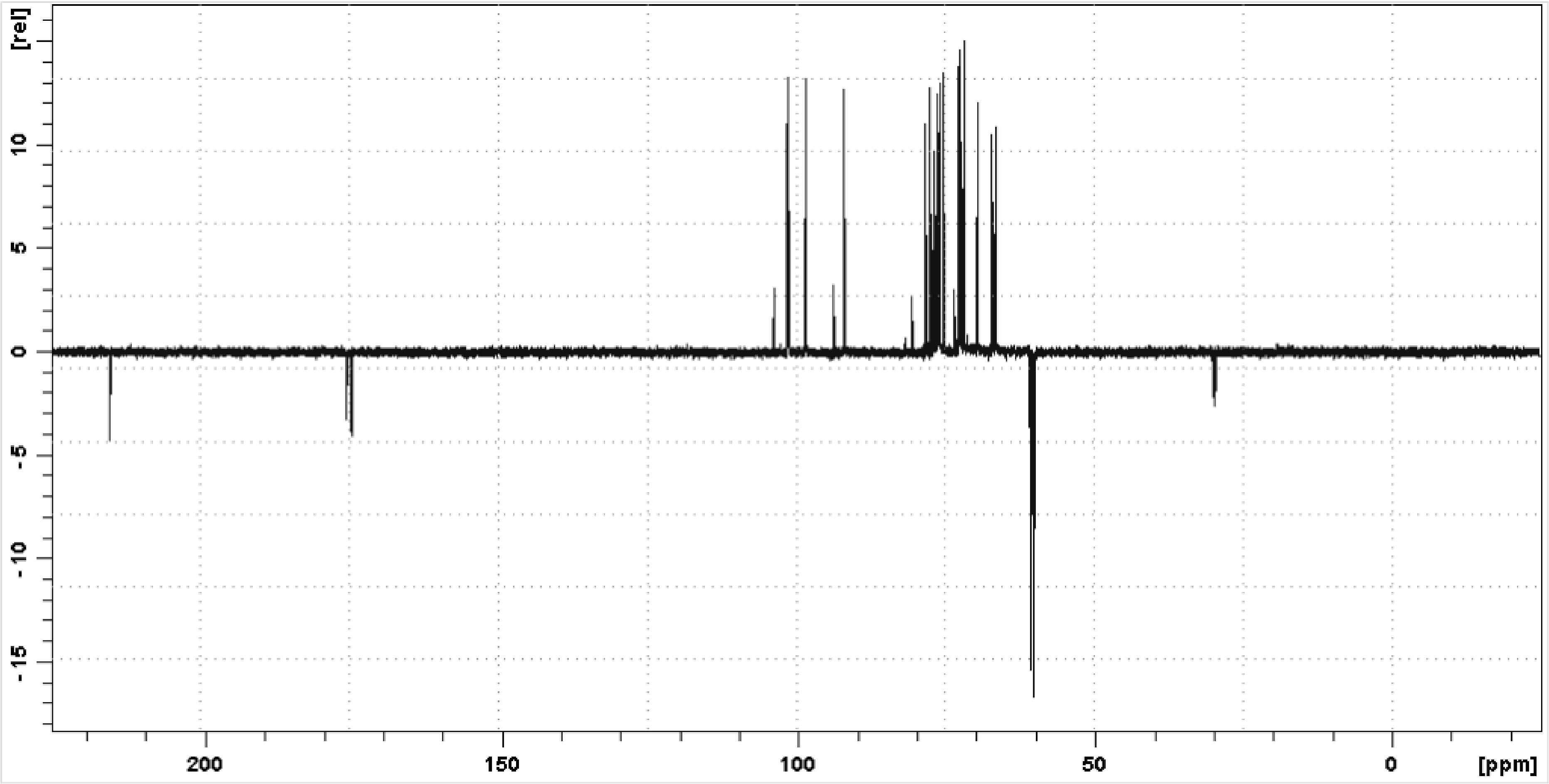

Figure S10. ^{1}H-NMR of **G3**.

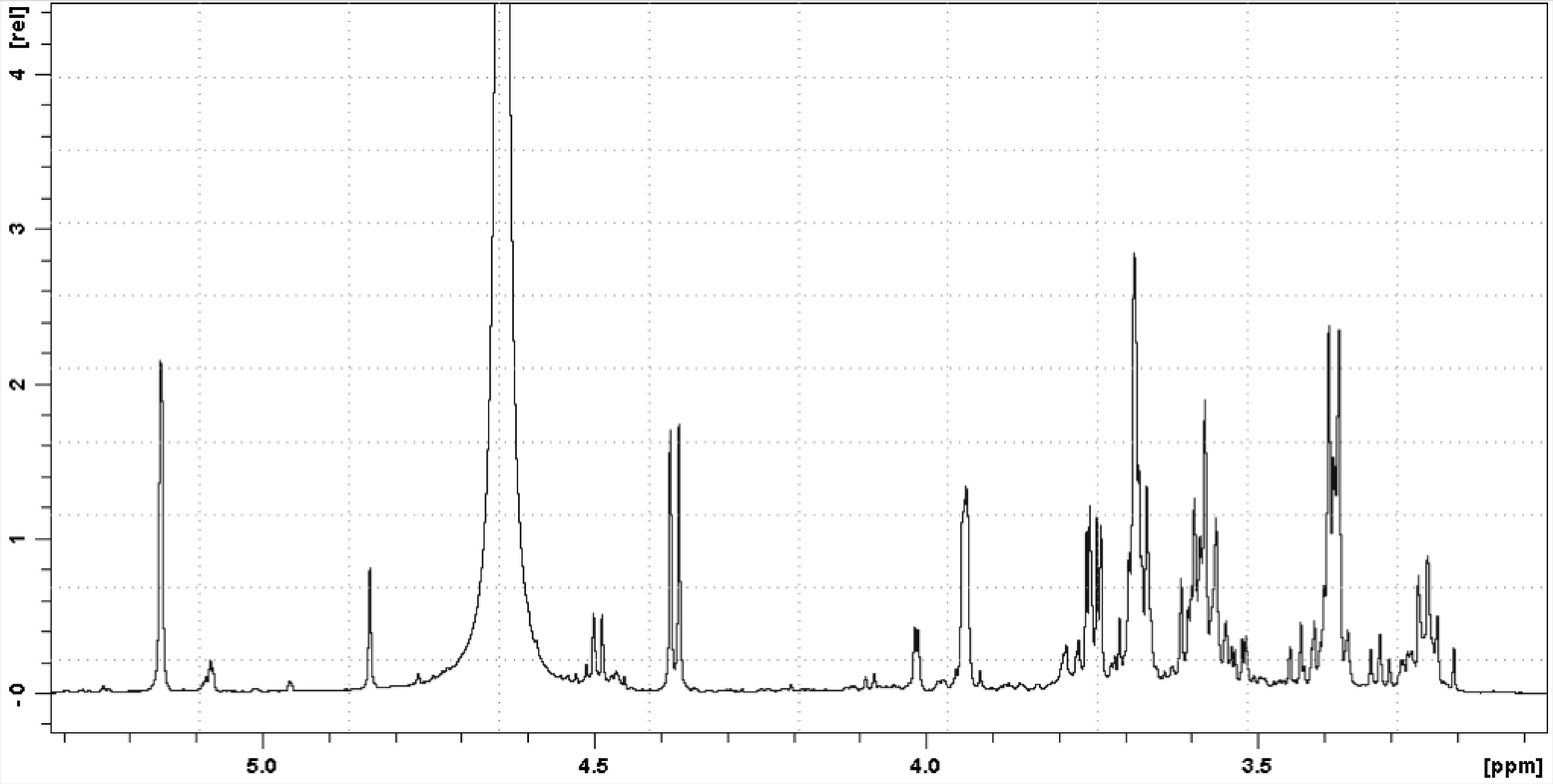

Figure S11. DEPTQ (C-NMR) of **G3**.

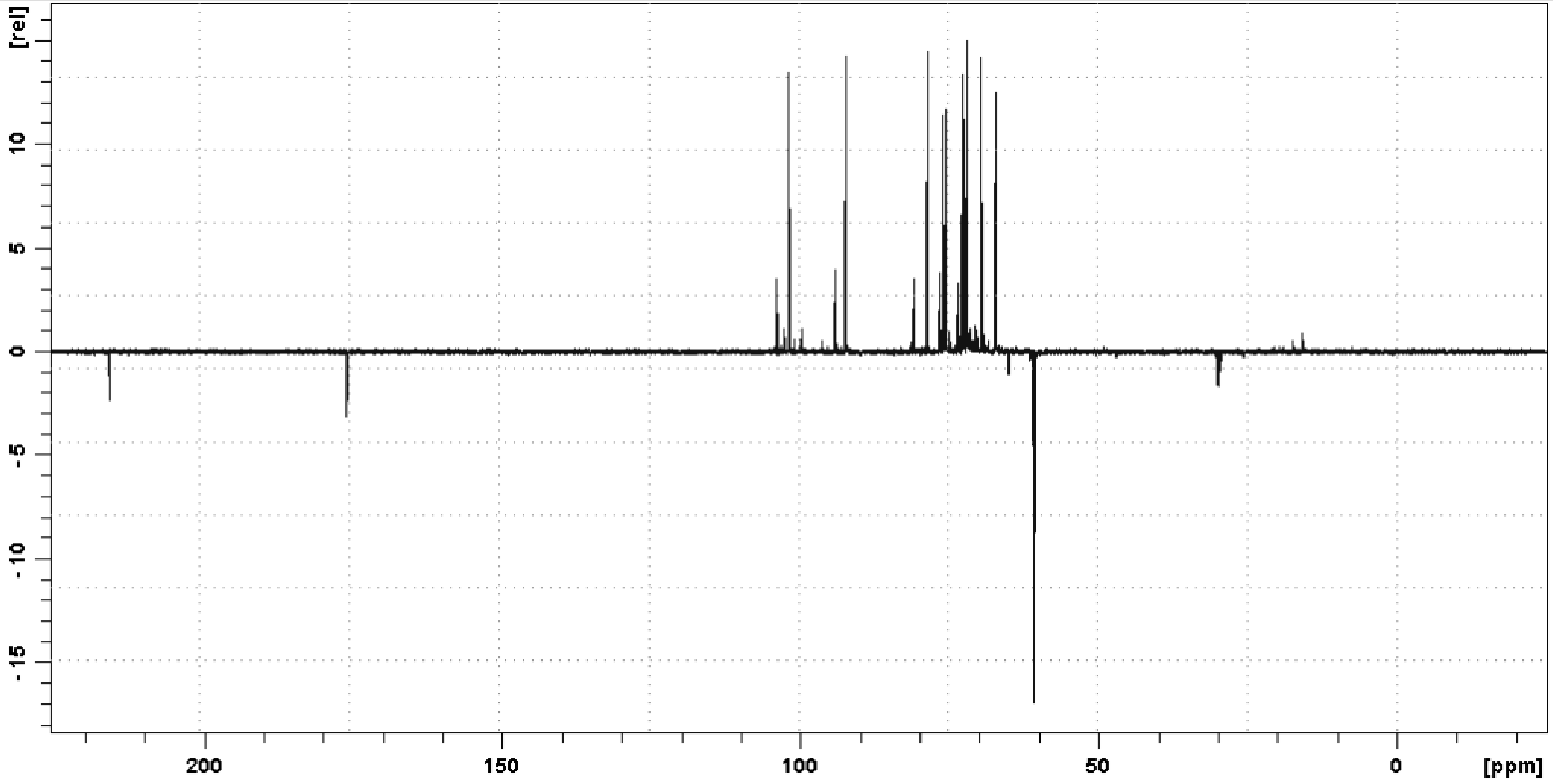

Figure S12. ^{1}H-NMR of **YT**.

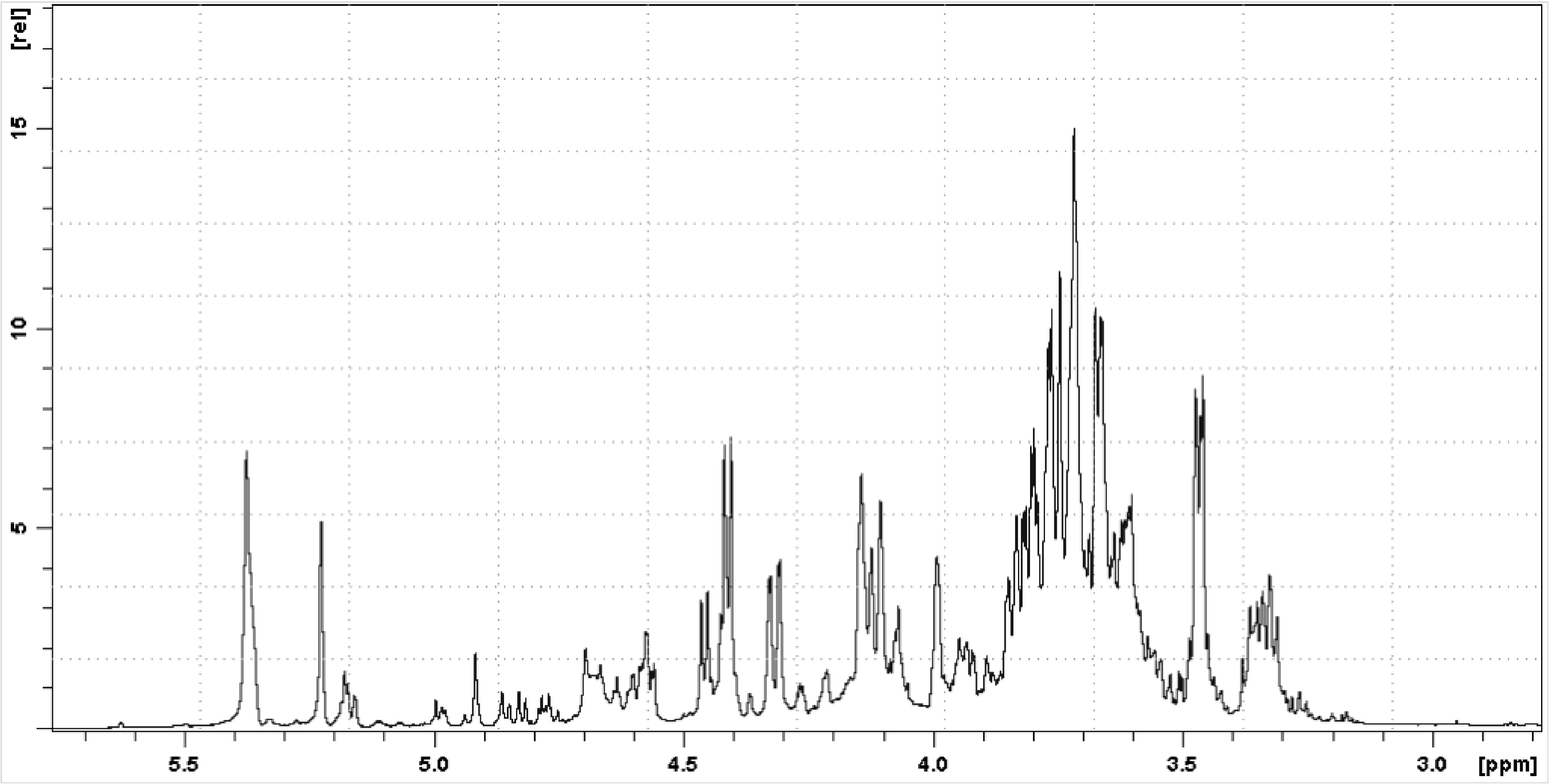

Figure S13. DEPTQ (C-NMR) of **YT**.

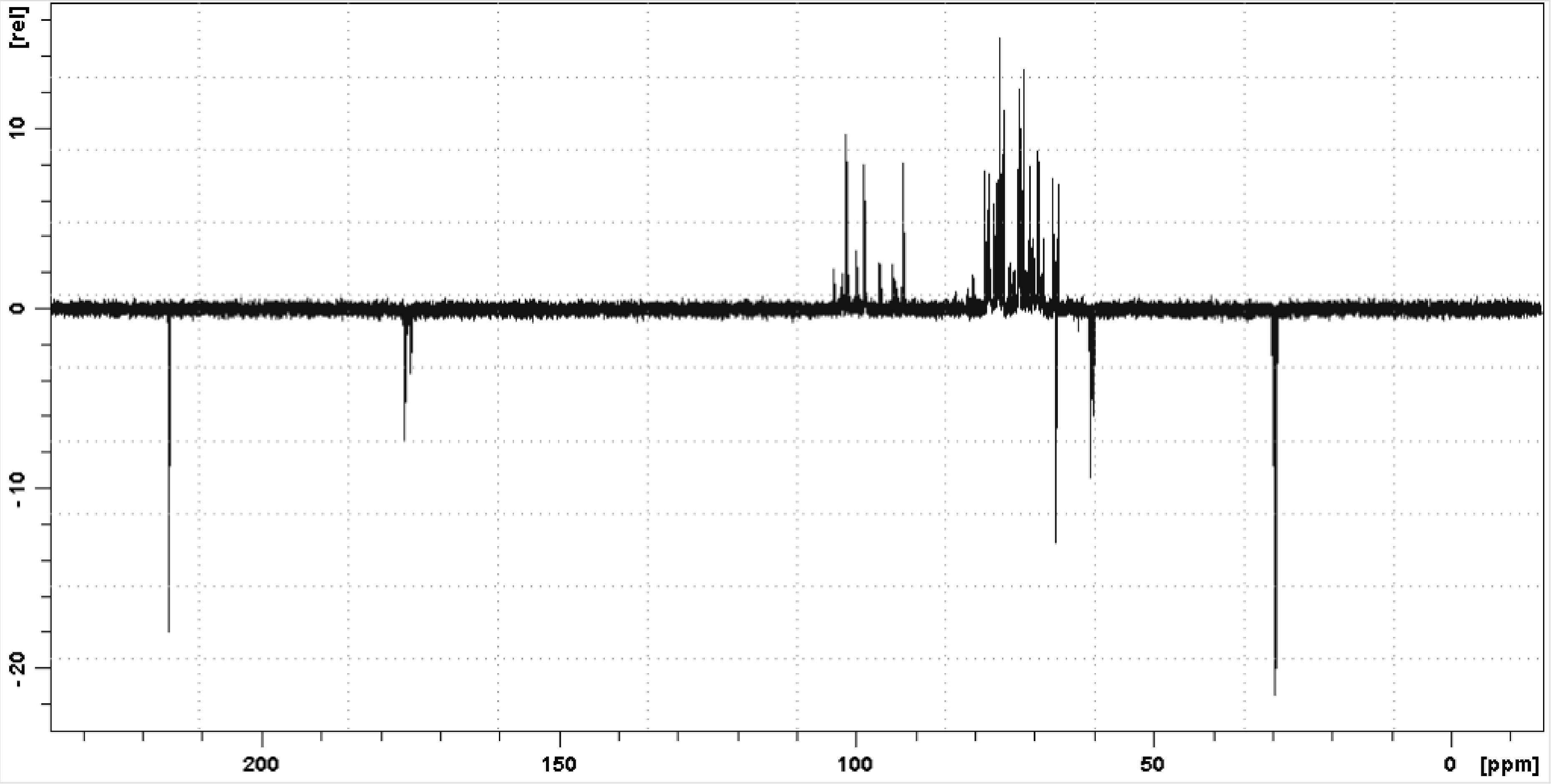

Supplement: Supplementary File 1: — PDF-Document (PDF, 473 KB) [file marinedrugs-10-02138-s001.pdf]
